# Method of Determining Sequence Actions of Products Improvement

**DOI:** 10.3390/ma15186321

**Published:** 2022-09-12

**Authors:** Andrzej Pacana, Dominika Siwiec

**Affiliations:** Faculty of Mechanical Engineering and Aeronautics, Rzeszow University of Technology, Al. Powstancow Warszawy 12, 35-959 Rzeszow, Poland

**Keywords:** quality, multi-criteria decision method, quality management tools, improving quality of products, Ishikawa diagram, DEMATEL method

## Abstract

Material production processes are special processes. As part of continuous improvement, it is extremely important to find the causes of the incompatibilities that occur in them. To increase the effectiveness of these actions, different methods are used. The purpose of this study was to present an original method that allows the classification to improve the combinations of actions of product with material incompatibility. The originality of this method allows for the sequential and coherent operation of adequate analysis techniques of causes resulting in incompatibilities in the product material and, consequently, identifying the reasons that influence their quality. The presented method was developed using a new combination of brainstorming (BM), the Ishikawa diagram with 5M rule, the DEMATEL method, and the algorithm used in the MATLAB software. As a result of the proposed applied method, it is possible to create a sequence of actions that include interactions between important causes of product incompatibility, which was supported by the test of this method. This method was shown to support the creation of a rank of importance of improvement actions. This ranking allows for improvement of any product according to the possibilities of enterprises and simultaneously allows for reducing or eliminating products’ incompatibilities.

## 1. Introduction

Taking action to improve products is the basis for developing companies and increasing customer satisfaction [1,2]. This improvement mainly includes stabilizing the production process of these products [3] and improving the quality materials of their formation [4]. The key is developing a possible comprehensive procedure of stability, i.e., methods, algorithms, and models. These procedures should have a universal function, i.e., for any products and incompatibilities identified in these [5,6,7]. Furthermore, it is necessary to use these procedures for any quality control, e.g., non-destructive testing (NDT) [8] or destructive testing (DT) [9]. The reason is that the choice of improvement actions does not only follow the expectations [10,11,12], but also from the quality control of these products and the incompatibilities detected there. After identifying the incompatibility, other techniques to determine a way to improve the product appear necessary.

The literature review was conducted, after which it was shown that the Ishikawa diagram was the main tool supporting the process of improving product quality. However, the study authors used the Ishikawa diagram, which is not usually used as a single effective tool. It was often combined with other techniques. For example, in work [13], a procedure is used to identify the root cause of casting of a product for a car. The Ishikawa diagram is used for that, which was combined with Pareto analysis to determine the number of occurrences of the causes of incompatibility. The same combination of quality management tools (i.e., Ishikawa diagram and Pareto analysis) was used by the authors of the studies, for example, [14,15]. Among other studies [14], the objective was to determine the concrete causes of sleeper defects in the production process and propose improvement actions. The authors of the study [15] used a combination with quality management tools to analyze the incompatibilities of welding seams. However, the authors of the study [16] used the Ishikawa diagram to analyze the causes of incompatibility and develop an experience matrix that supports the factorial experiment. The authors of studies [4,17] proposed improving the process of analysis of cause incompatibility, where quality management tools and fuzzy multi-criteria decision methods were integrated, that is, Ishikawa diagram, 5Why method, and FAHP method (Fuzzy Analytic Hierarchy Process) [17], or SMARTER method, brainstorming (BM), Ishikawa diagram, Likert scale validation technique, arithmetic average, and GRA method (Grey Relational Analysis) [4]. In turn, in the article [18], a combination of the Ishikawa diagram and Bowtie analysis was shown. The aim was to develop a method to visualize a gas turbine and to improve the risk assessment. Another example is a study [19] in which errors in assessment were in a variety of machine building scenarios. Three combined Ishikawa diagrams were used for it. Furthermore, the authors of the study [20] analyzed possibly using the combination of the 5Why method and the Ishikawa diagram to identify factors that impact production line downtime in the automotive industry. However, the study authors used the Ishikawa diagram [21] to reduce blow-off defects in the cast iron distribution box. In turn, the study authors [5] developed a model that supports the improved quality of industrial products. They used in this model, e.g., brainstorming (BM), Ishikawa diagram, and the 5Why method. The model test was carried out for the porosity cluster of the mechanical seal made of 410 alloy, where the defect was detected by the fluorescence method (FPI). The complex summary of the literature review is shown in Table 1.

After a literature review, it was concluded that the technique most commonly used to improve product quality was the Ishikawa diagram and its combinations with other techniques, mainly with Pareto–Lorenz analysis [13] and the 5Why method [5,17]. After these analyses, the main source cause of the problem was selected. Usually, this cause was in the area (category) of material of the product. In the study [2], the universal model was developed to predict the expected direction of improvement in product quality. In this were model techniques, i.e., SMARTER method [22], brainstorming method (BM), survey with the Likert scale, Weighted Sum Model (WSM), relative state scale, and the Naïve Bayesian Classifier (NBC). The concept of the model refers to determining the preferred quality of a product according to the importance of the product criteria and the evaluation of customer satisfaction from the states of the product criteria. Another example is the study [4] in which a new model was developed supporting the stability quality of materials and industrial products. The purpose of this study refers to analysis of the small number (even 4) of the incompatibility causes of product and determining the improvement actions with more precision and reducing subjective expert opinions. The quality management tools and multi-criteria decision methods were implemented in this model, i.e., SMARTER method, brainstorming (BM), Ishikawa diagram, validation technique with Likert scale, arithmetic average, and GRA method (Grey Relational Analysis). In addition, a pro-environmental method of sample size determination was developed to predict the quality level of the products considering current customers’ expectations [23]. This method was developed by modifying a procedure to determine the size of the research sample as part of the calculated estimator of the mean value in the general population. The method allows us to determine the number of potential customers (respondents) needed to provide product requirements, which were then processed and used to predict the quality level of the product. 

After comparing this study with the mentioned studies [2,4,23], it was concluded that the proposed method is complementary to previous methods and models that allows the improved quality of different products. In this case, the method is concentrated on eliminating the causes of incompatibility of products by identifying the ranking of improvement actions adequate to the main causes of these incompatibilities in the material area. Each of the previous studies did not focus on this problem. However, the solution obtained in this way does not take into account the possibility of taking alternative actions, e.g., resulting from the inability to implement an action eliminating the currently identified root cause in the area of materials. This was considered a research gap that was assumed to be filled.

Therefore, the purpose of the research was to develop a method that supports the classification of a combination of integrated product improvement actions in the context of material non-conformity. 

The originality of the study is to propose a new approach to analyzing the incompatibilities of products in the area of materials of the Ishikawa diagram, including rules of quality management methods and tools and multicriteria decision methods. The novelty is a sequential analysis of the incompatibility caused with the materials of products and their reduction to the main causes, i.e., influencing to the most degree the occurrence of product incompatibilities. In addition, a new method developed is developed which will support creating a sequence of integrated actions, including the interaction between important causes of product incompatibility in the material area. The newest is that this method supports creation of a sequence of actions that includes interactions between important causes of product incompatibility. The originality is also that this method supports creating a rank of importance of improvement actions. This ranking allows for the improvement of any product according to the possibilities of enterprises and simultaneously allows for reducing or eliminating products’ incompatibilities. This method will be useful for production companies in selecting the sequence of improvement actions based on their preferences (possibilities). In this context, the possibilities of the enterprise refer to current resources, for example, material, financial, and human. It is the possibility of taking improvement actions in a given period to eliminate the causes of non-compliance of the product. At the same time, the created ranking of the choice of improvement actions will allow reducing the effects influencing incompatibility of the products. Another originality is streamlining the use of the Ishikawa diagram by its combination with the multicriteria decision method, i.e., DEMATEL.

The method was tested based on a mechanical seal porosity cluster of the mechanical seal from 410 alloy. Incompatibility was detected by non-destructive testing in the Polish industry.

## 2. Method

### 2.1. General Concept of the Method and Choice of Tools

**Figure 1:**
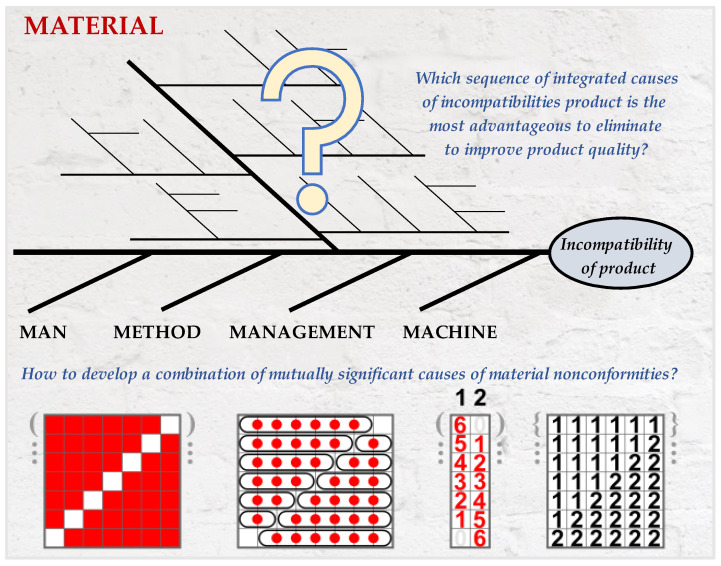
General concept of the method considering a process of the importance of improvement actions in materials of Ishikawa diagram.

The concept of the method refers to determining the integrated sequence of incompatibility causes of a product, which will simultaneously create a ranking of the combination of integrated improvement actions. These actions will be related to the area of the product material. The method used quality management tools and a multi-criteria decision method. The choice of methods and tools was based on a literature review, e.g., [4,5,13]. These techniques were as follows: SMARTER method [22], method of selecting a team of experts [5,11,23], brainstorming (BM) [5,24], Ishikawa diagram (fishbone, herringbone) [14,19,20], DEMATEL method [25,26,27,28], algorithm in the MATLAB program [7], and Pareto–Lorenz rule (20/80) [29]. The general concept of the method is shown in Figure 1. This Figure shows, in an intuitive way, the main concept of this method, e.g., the simplified diagram of Ishikawa, the idea of searching the combinations of causes of material incompatibility, and the main questions that occur during using the proposed method.

Initially, the main incompatibility of the product is determined (that is, generating the largest waste). Then, a team of experts is selected. This team will work according to the developed method. Then, the source cause of incompatibility is determined, so the place of occurrence of the incompatibility of product. Then, all causes of incompatibility of the product are generated, the so-called potential causes. Then, the categories of these causes are determined. The 5M rule is used for that, i.e., man, method, machine, material management. The potential causes are noted for these categories and then visualized in the Ishikawa diagram. Later, only the causes in the category “material” are analyzed. This results from a literature review, after which it was shown that most of incompatibility causes were identified in the “material” of the product. The second-order causes were determined only for causes from the “material” area. Then, the mutual impact of second-order causes was assessed. The DEMATEL method was used for that. After this method, the most important causes are determined, i.e., causes that have the most significant mutual influence on the occurrence of the incompatibility of the product. The integrated combinations of second-order causes are generated from these most important causes. The developed algorithm will be used for this, which is used, for example, in the MATLAB program. From these combinations a ranking will be developed, and it will be developed by calculating the impact of the integrated sequence (combination) of second-order causes on incompatibility. The higher the ranking value, the greater the influence of the integrated sequence of causes. Hence, it will be possible to determine which causes should be eliminated in the first order to improve the product. If any of the reasons for the incompatibility of the product in a given sequence cannot be eliminated, then on the basis of the prepared ranking, it will be possible to select another possible sequence. This procedure is favorable for the improvement of products according to a continuous improvement of their quality.

### 2.2. Assumptions of the Method

**Figure 2:**
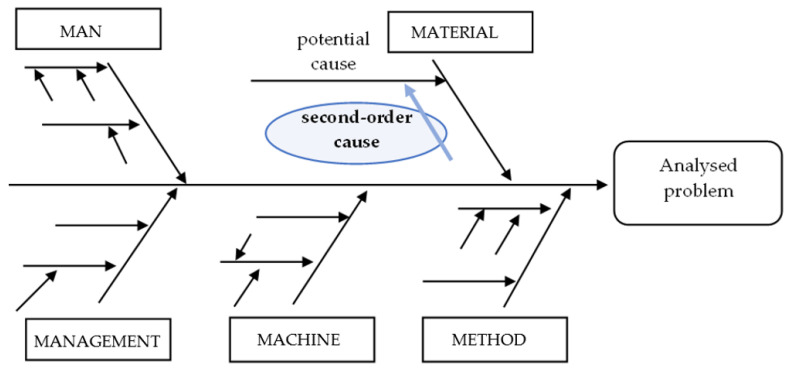
Example of kind of categories and way marked the causes on the Ishikawa diagram with 5M rule (i.e., man, method, material, machine, management).

The assumptions for the method were developed as part of the concept of the methods and in view of tools that support the realization of the method. These assumptions were selected after preliminary research and literature review, i.e.,

- the number of all potential causes of product incompatibility in the whole Ishikawa diagram is not limited [14,16,17];
- the number of potential causes determined in the category “material” in the Ishikawa diagram is not limited, but not less than 4 second-order causes (Figure 2) determined in this category [4];
- second-order causes are causes that influence the appearance of potential causes of product incompatibility and in this approach are determined only in the category ‘material’ [19];
- the important (main) causes are causes that have the greatest mutual impact on the occurrence of incompatibility and have values higher than the average value (α) [25,26,27,28];
- the integrated sequence of causes is determined only from the main causes belonging to the category (area) of “material”;
- the ranking of improvement actions results from the ranking of an integrated sequence of main causes belonging to the category “material” category [5,17];
- selecting the sequence (combination) of improvement actions, product results from the needs and predispositions of the entity using the method.

These assumptions are detailed in individual stages of the method, which are characterized in the next part of the study.

### 2.3. Description of the Method

**Figure 3:**
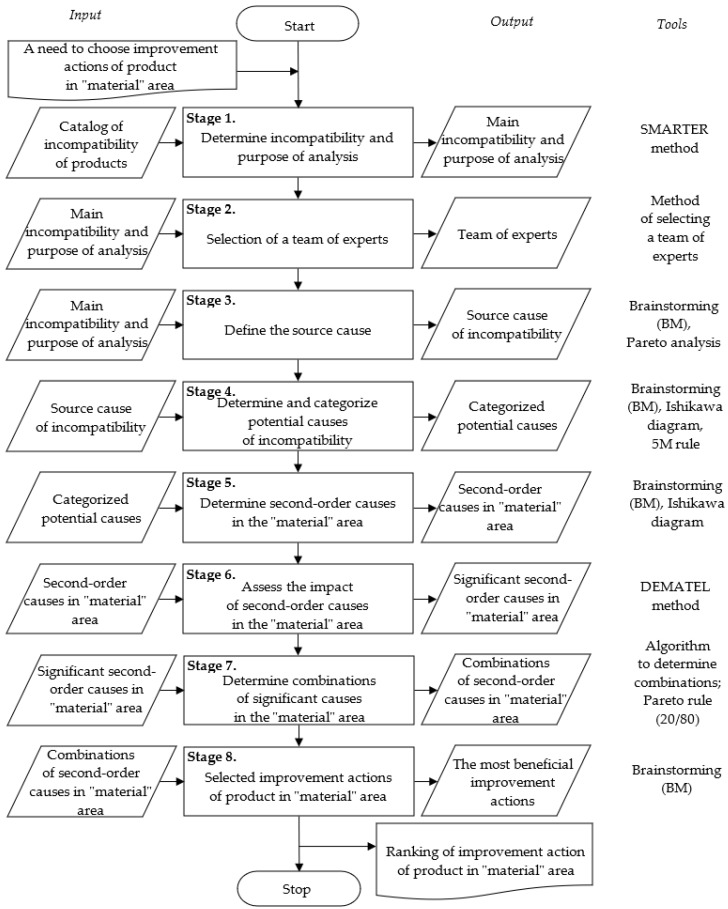
Algorithm of method of determining sequence actions of products improvement.

The method was developed in eight main stages. The description of these stages is shown in the next part of the study. However, the algorithm of the method is shown in Figure 3.

**Stage 1.** Determine the incompatibility and purpose of analysis.

Determining the incompatibility and purpose of the analysis is a task belonging to an entity expert, e.g., quality control manager. The incompatibility to analyze should be the main incompatibility, so the most occurring. To choose incompatibility, it is possible to use control sheets or Pareto–Lorenz analysis [29]. Then, the purpose of the analysis is determined according to this incompatibility. The SMARTER method can be useful for that [22]. The purpose should include the elements that characterize the product, e.g., kind of incompatibility, material of the product, and number of occurrences of incompatibility. In an analysis of the problem, it will be necessary to consider the properties of the materials, as shown, for example, in the study [30].

**Stage 2.** Selection of a team of experts

The selection of a team of experts is necessary for effective achieving the purpose of analysis by using the method (determined in the first stage). Therefore, the team of experts should have knowledge and experience in analyzing selected incompatibility, teamwork skills, and competencies to achieve the purpose of the analysis. The choice of expert team is made according to methods shown in studies, e.g., [5,23].

**Stage 3.** Define the source cause

Defining the source cause relies on determining the place in which the incompatibility of products occurs. To this end, the brainstorming method (BM) [5,24] or the 5Why method (Why-Why) [4] can be used. 

**Stage 4.** Determine and categorize the potential causes of incompatibility

The potential causes are causes which probably have an impact on the occurrence of the incompatibility of products. The potential causes are generated by answering the question “What has happened that incompatibility occurred?”. It is necessary to determine all potential causes according to the source cause. For this purpose, brainstorming (BM) is used by a team of experts, but not for more than 30 min. The causes should be noted in a place visible to the team of experts, e.g., a table. Later, the real causes should be removed, i.e., causes that have negligible impact on the occurrence of incompatibility. Other potential causes should be grouped for their standardized analysis. The Ishikawa diagram with the 5M rule is used for it, that is, the method of material man, management, and machine method [14,19,20].

The Ishikawa diagram (herringbone, fishbone) was first developed for all 5M categories, as shown in studies, e.g., [4,17]. The purpose is to separate the potential causes belonging to the “material” area from the other causes generated during brainstorming. Therefore, all potential causes should be listed in the appropriate category of 5M. The decision about the category of cause is made by a team of experts and then this cause is noted on the Ishikawa diagram [4,5,17]. 

**Stage 5.** Determine second-order causes in the “material” area

It was assumed that the method will support the ranking of combinations of integrated improvement actions in the “material” area. Therefore, a detailed analysis is realized only for the causes of incompatibility included in the “material” category of the Ishikawa diagram. First, it is necessary to analyze potential causes in the “material” category to determine second-order causes, i.e., causes occurrence and potential causes. Second-order causes are determined by the team of experts during brainstorming (BM) by answering the question “What has happened that potential causes occurred?”. All second-order causes are noted in the Ishikawa diagram for the appropriate potential cause. The process of detailing potential causes by second-order causes should end after 30 min. The minimum number of all second-order causes in the “material” area is equal to 4 [4].

**Stage 6.** Assess the impact of second-order causes in the “material” area

The evaluation of the impact of second-order causes is performed by a team of experts using the DEMATEL method [25,26,27]. The team evaluated all causes on a multilevel ordinal scale of direct influence (0–4) [26], i.e., 0—no impact, 1—low impact, 2—clear impact, 3—high impact, 4—extreme impact [26,27]. The assessments are taken separately by each team expert. The median is calculated from the evaluations of all experts [31]. Based on the median of the evaluation of the influence of the given elements on each other, a direct influence matrix is created, where there are zero values on the diagonal (no influence of identical elements on each other) (1) [28]:

(1) $$z_{\mathrm{ij}}=\frac{1}{l}\overset{l}{\underset{k=1}{\sum }}a_{\mathrm{ij}}^{k},$$

where: $a_{\mathrm{ij}}^{k}—$rating, l—opinion, i,j=1,2, …, n.

According to the direct influence matrix, it is possible to create a network of connections (interactions), the so-called direct influence structure [27]. On the basis of this, the indirect influence matrix (structure) is created, so the influences are exerted by other elements of the system. It is a normalized matrix of direct influence $\left(X={\left[x_{ij}\right]}_{n\times n}\right)$, as in Formula (2) [26]:

(2) $$X=\frac{Z}{s}\;\;\;\;\mathrm{where}:\;s=\max\left(\underset{1\leq i\leq n}{\max}\overset{n}{\underset{j=1}{\sum }}z_{\mathrm{ij}},\;\underset{1\leq i\leq n}{\max}\overset{n}{\underset{i=1}{\sum }}z_{\mathrm{ij}}\right)$$

where: all elements of the X matrix are in range 0 $\leq x_{ij}\leq 1,$ 0 $\leq \sum_{j=1}^{n}x_{ij}\leq 1,$ and the last element i is shown as $\sum_{j=1}^{n}z_{\mathrm{ij}}\leq s$. Then, the total influence structure is created. Direct and indirect influences are simultaneously included in this structure. It is the sum of direct and indirect effects, that is, (3) [26,27]:

(3) $$T=X+X^{2}+X^{3}+\ldots +X^{h}=X{\left(I-X\right)}^{-1},\;\;\;\;\mathrm{when}\;h\to \infty$$

where: X—normalized matrix of direct influence, I—identical matrix.

The dependencies of the causes’ effects are determined based on this structure, as shown in Formula (4) [26,27]:

(4) $$\begin{array}{cc} R={\left[r_{i}\right]}_{n\times 1}={\left[\overset{n}{\underset{j=1}{\sum }}t_{\mathrm{ij}}\right]}_{n\times 1} & C={\left[c_{j}\right]}_{1\times n}={\left[\overset{n}{\underset{i=1}{\sum }}t_{\mathrm{ij}}\right]}_{1\times n}^{T} \end{array}$$

where: R—sum of values in the rows of the total impact matrix, C—sum of the values in the columns of the total impact matrix, r—the sum of the *i*-th row in the T matrix and determines the sum of direct and indirect effects not included among the verified elements, c—the sum of the *j*-th column in the T matrix and defines the sum of direct and indirect effects not included among the verified elements.

Next, the average value (α) is calculated, which determines the important mutual impact of second-order causes on the appearance of incompatibility of the product (5) [25,27]:

(5) $$\alpha =\frac{\sum_{i=1}^{n}\sum_{j=1}^{n}\left[t_{ij}\right]}{N}$$

where: as in Formula (7).

The values of the T matrix above average (α) determine the significant mutual influence of second-order causes on the appearance of the product. Causes less than the average value (α) were omitted in further analysis because these causes do not have a significant impact on the appearance of product incompatibility.

**Stage 7.** Determine combinations of significant causes in the “material” area

At this stage, combinations of second-order causes that have a significant mutual impact on the product occurrence of the incompatibility are determined. These causes are determined only for second-order causes determined in the “material” area. It is necessary to determine all combinations of integrated causes of incompatibility of the product. If the number of causes is large, it is proposed to use computer software, e.g., the MATLAB program. It is possible to base it on the formula developed for use in this program, i.e., (6) [7]:

(6) $$F=\left[u_{i},\ldots ,u_{n}\right]\;M=nchoosek\left(l:n,k\right)$$

where: *u*—cause, which has a significant mutual impact on the occurrence of the incompatibility, *l*—the number of the reason from which the sequence should be determined, *n*—the number of the reason for determining the sequence, *k*—the number of causes integrated in one combination, *i*—1, 2, …, *n*.

It is proposed to assume that the number of *k* causes integrated in one combination will correspond to 20% (in the Pareto principle) of all causes having a significant mutual influence on the appearance of nonconformity (*u*). It results from the Pareto–Lorenz principle (20/80), where improvement actions should initially focus on only a few most important causes of incompatibility and the remaining ones should be reduced sequentially [29]. Based on the determined combinations of integrated significant causes of nonconformity, a ranking of improvement actions is created, as presented in the next stage of the method.

**Stage 8.** Selected improvement actions of the product in the “material” area

Improvement actions are determined according to the combination of significant integrated causes of incompatibility. For this purpose, the calculated weight of the given combination should be taken. It is the product of the cause weights for a given combination divided by the number of causes included in this combination, as shown in Formula (7):

(7) $$w_{i}=\frac{x_{ij}\times \ldots \times x_{n}}{k}$$

where: *x*—weight of the *i*-th cause for the *j*-th combination, *k*—number of causes in a given combination, *i*, *j*, *n* = 1, 2,…, *m*.

The weights of causes ($x_{ij}$) were calculated in the seventh stage of the method in the total impact. The weight of causes is on the diagonal of this matrix. After calculating the weights, it is possible to rank combinations of integrated improvement actions in the “material” area, where these combinations will contribute to reducing the effects of incompatibility in the analyzed product. It relies on developing a ranking from weights of combinations ($w_{i}$), i.e., from the maximum weight to minimum weight. The maximum weight refers to the combination of causes that should be eliminated or reduced first. However, in case of a lack of possibilities to take action on improvement, even for one cause in the combination, it is necessary to verify the combination that is the next in the ranking. If the weights of the combinations are the same (or differ slightly from each other), the choice of combination is not limited. The final decision belongs to the entity (expert) and depends on the possibilities of the enterprise and simultaneously on the possibility of reducing the effects of incompatibility of the product.

## 3. Test of Method

A method based on a porosity cluster on a mechanical seal of the 410 alloy was tested according to the concept of the method and the algorithm, that is, in eight main stages. 

**Stage 1.** Determined incompatibility and purpose of analysis

**Figure 4:**
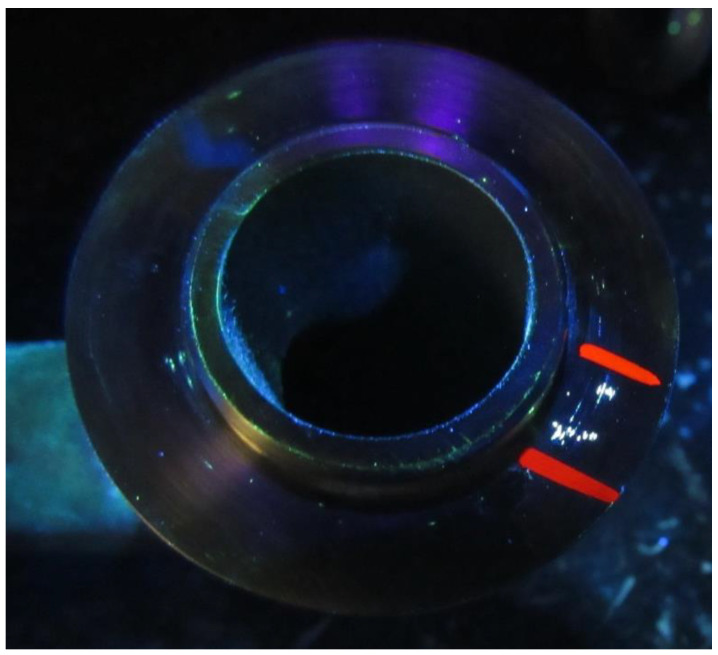
Example of the mechanical seal with porosity cluster.

The incompatibility for the analysis was the porosity cluster with the mechanical seal of the 410 alloy. The choice of the mechanical seal of the 410 alloy was based on the individual needs of the company. This is the product of the new generation and, hence, its quality in this company was not stabilized. This product is popular and often used, e.g., in the engineering industry. The results obtained for the mechanical seal can be useful in different applications. It was the main incompatibility (which occurs a lot) in the Polish production company. The porosity cluster (that is, the microporosity of the product) was not visible to the naked eye. However, quality control of the products was realized. The mentioned incompatibility was detected by non-destructive testing (NDT), that is, the fluorescent method (FPI), as shown in studies [5,32]. The porosity cluster was small, which disqualifies the product for production. Detailed characteristics of this type of incompatibility are shown in Ref. [5]. In view of this, the developed method was used to carry out adequate improvement actions. An example of a mechanical seal with a porosity cluster is shown in Figure 4.

A mechanical seal is a cast and welded product. This type of seal is a new-generation seal, that is, it allows reducing leaks and damage to the shaft or pump sleeve, but it also allows the product to be sealed in a vacuum manner. Additionally, it has self-regulating features of disc wear, minimizes the possibility of bearing contamination, and protects products against damage [5,32,33]. The mechanical seal was made of alloy 410, i.e., martensitic and stainless steel. Alloy 410 allows the product to maintain high strength and pressure resistance. Physical, mechanical, and chemical properties of alloy 410 are presented in [5].

After determining the incompatibility of the product, it is possible to determine the purpose of the analysis. The purpose was to rank combinations of integrated improvement actions in the “material” area, where these combinations will allow a reduction of the effects of the porosity cluster on the mechanical seal of 140 alloy.

**Stage 2.** Selected by a team of experts

In the second stage of the method, a team of experts was selected. The choice was made according to the proposed method. Therefore, it was done as shown in studies [5,23]. The team of experts was selected: quality control manager, quality control employee, and article authors. The team of experts had knowledge and experience in the analysis of these types of incompatibilities. Additionally, they were characterized by teamwork skills and had competencies that supported the achievement of the analysis goal.

**Stage 3.** Defined source cause

Then, the source cause of the porosity cluster on the mechanical seal was defined during brainstorming (BM) carried out among a team of experts. It was concluded that the root (place) of this incompatibility was a gas that dissolves in liquid metal. This is because of the ability to dissolve gases at lower temperatures of the metal. Then, these gases are separated from the solution and trapped in the metal during its solidification, as also confirmed by the authors of the studies [5,34].

**Stage 4.** Determined and categorized potential causes of incompatibility

In the fourth stage of the method, the team of experts determined the potential causes of the cluster in the mechanical seal of the 410 alloy. For this, the brainstorming method (BM) was used. The causes were generated by answering the question “What happened to create a cluster of porosity?”. The causes were determined according to the source cause, i.e., gas that dissolves in liquid metal. The brainstorming ended after 30 min. As a result, potential causes were generated, i.e.,

1. lack of up-to-date procedures and instructions;
2. significant nitrogen and hydrogen content in the arc atmosphere;
3. incorrectly selected gas shield;
4. electrode moisture build-up;
5. high coagulation rate;
6. metallurgical reactions arising in cuffed gas;
7. release of carbon monoxide and carbon dioxide;
8. moisture in the flux is caused by automatic welding;
9. rust formation on the welding wire;
10. employee mistakes;
11. little employee experience;
12. psychophysical state of the employee (e.g., severe nervous tension, exhaustion, physical or mental malaise);
13. inappropriate pace of work;
14. no mandatory refresher training;
15. lack of directional solidification due to a faulty casting system;
16. no TMP (Total Productive Maintenance);
17. sand in the molding mass;
18. inadequate supervision of production;
19. water in the molding sand;
20. problematic working conditions, e.g., noise, poor lighting, and room temperature.

**Figure 5:**
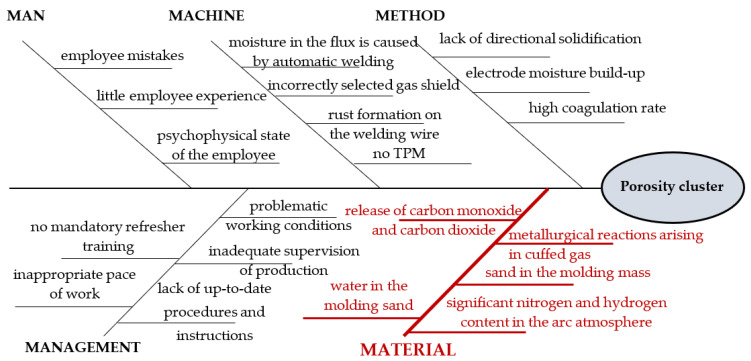
Ishikawa diagram for porosity cluster on mechanical seal.

All reasons were considered real to occur. Therefore, all potential causes were further analyzed. At this stage of the method, the potential causes were grouped according to the 5M formula and visualized on the Ishikawa diagram. The result is shown in Figure 5.

In this approach, the method supports the ordering of integrated combinations of improvement actions in the “material” area; therefore, the causes included in this category were analyzed.

**Stage 5.** Determined second-order causes in the “material” area

**Figure 6:**
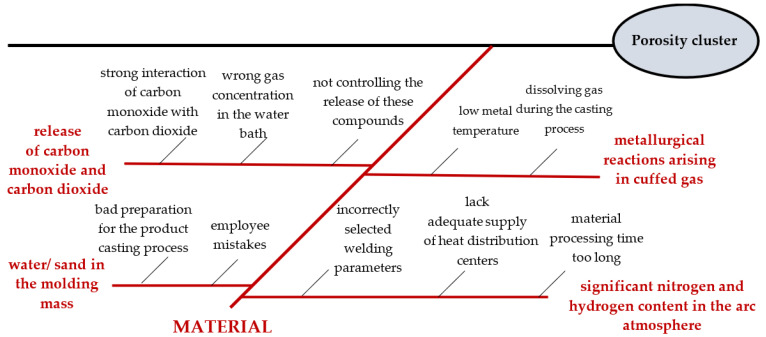
Fragment of the Ishikawa diagram for the “material” category.

The purpose of this stage was to determine second-order causes, so causes occur as potential causes. These causes were determined by a team of experts during brainstorming. When determining them, an answer was given to the question “What happened that the potential cause occurred?”. After about 30 min, the process of describing the potential causes in the “material” area was completed (Figure 6).

Potential causes, i.e., water in the molding mass, were considered as one potential cause. This was due to the same second-order causes identified for them. For each potential cause, at least two second-order causes were generated. The number of all second-order causes in the “material” area was greater than 4. Therefore, it was possible to implement the next stage of the method.

**Stage 6.** Assessed impact of second-order causes in the “material” area

The team of experts evaluated the mutual impact of second-order causes on the appearance of porosity clusters on the mechanical seal. According to the concept of the method, the evaluations were performed on a scale of 0 to 4. The median of these assessments was calculated. Based on the median and Formula (1), the direct impact matrix was created. The results are shown in Table 2. 

Based on the direct impact matrix, the indirect impact was created. It is a normalized direct impact matrix, which was created according to formula (2). The results are shown in Table 3.

Based on the values of the normalized matrix, the structure of the total impact was created. It was the sum of direct and indirect effects, as shown in Formula (3). The results are shown in Table 4.

Based on the structure of the total impact, the dependencies of the causes’ effects and the average values (α) were calculated. The average value determined a significant mutual influence of second-order causes on the occurrence of a porosity cluster on the mechanical seal. Formulas (4) and (5) were used for that. The results are shown in Table 5.

The second-order causes of the formation of the mechanical seal porosity cluster were: incorrect gas concentration in the water bath (A1), strong interaction of carbon monoxide with carbon dioxide (A2); failure to control the release of these compounds (A3), dissolution of gas during the casting process (A5), lack of adequate supply of heat distribution centers (A8) and material processing time too long (A10). They were caused by the following reasons: low metal temperature (A4), employee errors (A6), poor preparation for the product casting process (A7), and incorrectly selected welding parameters (A9). However, after using Formula (5), the average value was calculated, i.e., α = 0.77. The values of the total impact matrix above the mean value (α) characterized the causes that had a significant mutual influence on the appearance of the mechanical seal porosity cluster (Table 6).

**Figure 7:**
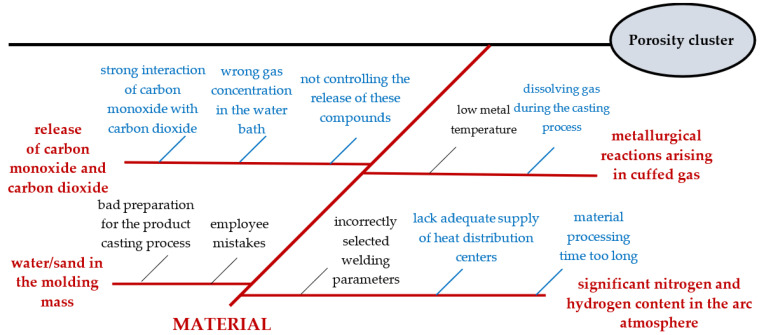
Result of the DEMATEL analysis combined with the Ishikawa diagram.

It was shown to lack a significant impact on causes, i.e., low metal temperature (A4), employee mistakes (A6), poor preparation for the product casting process (A7), and incorrectly selected welding parameters (A9). Therefore, these reasons were omitted from the further analysis. The causes analyzed were marked on the Ishikawa diagram and weights were assigned according to the results of DEMATEL (Figure 7).

According to the results from DEMATEL analysis combined with the Ishikawa diagram for the “material” area, this was further verification of the porosity cluster on the mechanical seal.

**Stage 7.** Determined combinations of significant causes in the “material” area

Based on the significant causes of the clustering of porosities in the mechanical seal, the combinations of these causes were determined. There were causes in the “material” area. The algorithm developed in the MATLAB program was used to determine all combinations (that is, Formula (6)). The number of significant causes was equal to 6 (i.e., A1 = 1, A2 = 2, A3 = 3, A5 = 4, A8 = 5, A10 = 6). According to the Pareto rule (20/80), the *k* causes were integrated into a single combination, where *k* was equal to 2 causes, as shown in Formula (8):

(8) $$F=\left[1,2,3,4,5,6\right]\;M=nchoosek\left(1:6,2\right)$$

According to the algorithm, 15 possible combinations of significant integrated causes of the porosity cluster on the mechanical seal were achieved. For each cause of the combination, a weighting was assigned, which was calculated in Step 6 of the method (that is, in the structure of the total impact). The result is presented in Table 7.

**Stage 8.** Selected improvement actions of the product in the “material” area

Based on the combination of significant integrated causes of incompatibility, improvement actions were determined. The weights for all 15 combinations were calculated. Formula (7) was used for that. Then, the combinations of integrated improvement actions were ranked in the “material” area, where these combinations will help reduce the effects of a porosity cluster on the mechanical seal. The results are shown in Table 8.

It has been shown that for a combination 10, 2, or 12, the first decision can be made to introduce improvement actions that will reduce or eliminate the causes of the porosity cluster. The decision is made by the entity (expert, e.g., company manager). If no improvement actions can be taken for the causes of these sequences, the second-ranked combinations should be verified. In this way, it is possible to make dynamic decisions as part of the continuous improvement of the mechanical seal where the porosity cluster has been identified. 

## 4. Discussion

Taking improvement actions in the area of product materials is still a challenge [35,36,37]. Appropriate procedures are sought to support dynamic decision-making in this regard [38,39,40,41,42], mainly in view of the need to meet customer expectations while coexisting with enterprise restrictions. Therefore, the objective of the study was to develop a method that supports the classification of actions to improve a combination of integrated products in the context of nonconformities of materials. The novelty of the incompatibility analysis study was to improve the process dedicated to the material area considering the rules of the quality management tools and decision methods. The proposed method is a new approach to analyzing the incompatibility of causes in the material area and their sequence of reducing and integrating to develop integrated sequences of improvement actions. The developed method, with certain assumptions made, can also be used in other areas of the Ishikawa diagram and in other analyses.

The method test was carried out on a mechanical seal. In this product, the porosity cluster was detected, which was identified by non-destructive tests in the Polish industry. First, the incompatibility for analysis and purpose was selected. The SMARTER method was used for that. Then, a team of experts was selected using a dedicated method. Then, during brainstorming (BM), the source cause of the porosity cluster on the mechanical seal was defined. It was shown that the sources (places) of this incompatibility were gases that dissolve in the liquid metal. Continuing with BM, potential causes were identified. All generated causes were equal to 20. The Ishikawa diagram with the 5M rule was used to group and visualize. Subsequently, the second-order causes belonging to the “material” area were determined and then they were assessed using the DEMATEL method. The result was the most significant cause, that is, causes that had a significant mutual impact on the porosity cluster. These causes were verified in the next stage, where their combinations were determined. These combinations were determined according to the algorithm developed that is applicable in MATLAB and according to the Pareto principle (20/80). As a result, 15 combinations were identified, on the basis of which the so-called importance of the combination of integrated causes of incompatibility was identified. On the basis of the values of these weights, a ranking was developed to take improvement actions in the area of materials. Therefore, it was shown that determining the importance rank of the integrated sequence of improvement actions, which will result from product incompatibility caused in the materials area of the Ishikawa diagram, allows for improved products according to the possibilities of enterprises, simultaneously reducing or eliminating the incompatibilities of products.

**Figure 8:**
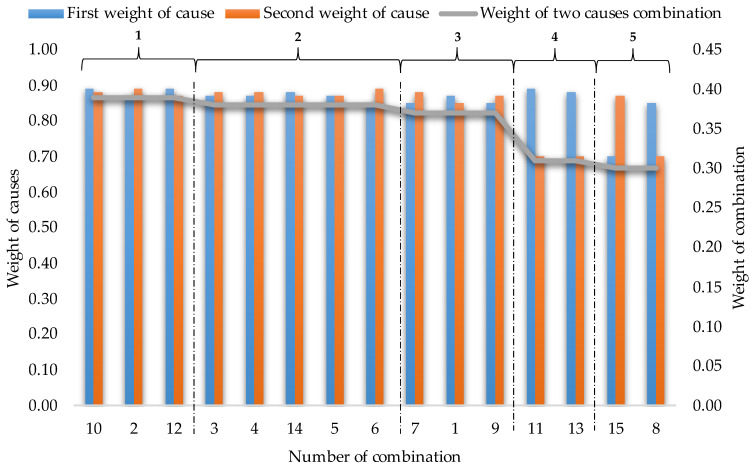
Comparison results of analysis.

The traditional approach to the analysis of the causes of incompatibility of products was compared with the approach proposed in this study. Previous analyses did not include the importance of causes [13,16]. Heuristic techniques were used for that [17,21]. The main cause was selected by a team of experts. For this reason, improvement actions were taken [4,15]. It was concluded that the traditional way is subjective and inaccurate [4,18]. Whereas this method supports the choice of improvement actions for causes, the most influencing on occurs the incompatibility. This is realized in a calculation way that is extended by the multicriteria decision method. In addition, in this method, the weights of causes are combined in different combinations (Figure 8).

It was observed that even small differences in the weight (importance) of the causes could affect the effectiveness of improving the actions. Additionally, combining these causes in combination allows for a choice of improvement actions simultaneously in view of their effectiveness and enterprise possibilities (e.g., financial resources). This contributes to increasing the accuracy and objectivity of decisions. In this case, there are three combinations, with the most effective to eliminate and reduce incompatibility. If no action is initiated for the first group of combinations, then the possibility of taking action for the cause combination in the second group should be explored. It was observed that the differences are slight between the first and second group of combinations. Therefore, their removal may to a similar extent improve the quality of the product. In turn, the final decision may be conditioned by other factors (individual company preferences). On the other hand, the fourth and fifth groups of combinations are the least desirable, i.e., the least important for improving the quality of the product. Therefore, the causes in these groups should be eliminated last, or they can be omitted. Hence, the proposed method supports dynamics making decisions about improving actions products in the material area, where these decisions will be made, e.g., by production enterprises in view of current resources and possibilities, taking into account the aspects of reducing the effects of incompatibility as much as possible. The method can be used by any company to improve any products and to analyze the causes of each type of non-compliance on them.

The main benefits of the proposed method include:

- making it effective and possible to realize decisions about the actions of product quality;
- sequence and coherent analysis of product incompatibility, which is supported by the calculating process;
- the right selection of improvement actions that limit the waste of resources;
- possible to analyze any products and their incompatibility;
- low-cost but effective method supporting the taking of actions and correct decisions in the product improvement cycle;
- choice sequence (combination) of the improvement actions of the product resulting from the needs and predisposition of an entity using this method.

However, limitations of the method include, for example, the dependence of the effectiveness of the method on the knowledge and experience of a team of experts, and the need to perform complex calculations.

This study has the objective of showing that it is possible to develop a method that supports the process of determining the rank of improvement actions. The carried test shows the practical possibility of using this method. This test was concentrated on the “material” area of the Ishikawa diagram (product quality in the context of material). However, under certain assumptions, the method developed can also be used in other areas of this diagram. The tools used in the proposed method have been universal applied, so it could be used separately use to analyze the incompatibility causes of products and to support making decisions in this area. Carrying out more tests in the future can also contribute to the probability and highlight advantages of methodic testing and alternatively show its limitations. They could also contribute to lifting effectiveness of methodic in case of the specification of process in which it will be used. In addition, future research will be based on developing the dynamic platform of this method. This platform was created as part of information about various incompatibilities that occur with different types of products. Furthermore, as part of future research, it was assumed that the effectiveness of different combinations of improvement actions was compared. This platform will be developed in the form of a computer tool.

## 5. Conclusions

Improving the quality of products mainly concerns the stabilization of the production processes of these products and the improvement of the quality of the materials made from them. However, it is still a challenge. Therefore, the objective of the study was to develop a method that supports the classification of actions to improve a combination of integrated products in the context of nonconformities of materials. In this method, quality management tools and the multicriteria decision method were used, i.e., SMARTER method, method of selecting a team of experts, brainstorming (BM), Ishikawa diagram with the 5M rule, DEMATEL method, algorithm in the MATLAB program, and Pareto rule. The method test was carried out for the porosity cluster on the mechanical seal of the 410 alloy. After testing the method, it was shown that determining the rank of importance of the integrated sequence of improvement actions, which will result from the product incompatibility caused in the materials area of the Ishikawa diagram, allows for improved products according to the possibilities of enterprises, simultaneously reducing or eliminating the incompatibilities of the product. Additionally, the method has managerial implications, e.g., increased customer satisfaction by improving the quality of the product material, the possibility of achieving better economic results by meeting customer expectations and improving the product by eliminating the main causes of incompatibility, and low-cost tool supporting activities reducing the waste of resources (MUDA).

## Figures and Tables

**Table 1:** Complex summary of literature review.

Example of Literature Position	Purpose of Study	Tools
[5]	universal model supporting improvement industrial products considering traditional quality management tools, where this model supports analysis of incompatibility causes and identification of the improvement actions	brainstorming (BM), Ishikawa diagram, the 5Why method
[4]	analysis of the small number of incompatibility causes of product and determining the improvement actions in more precision way, and reducing subjective of experts’ opinion	SMARTER method, brainstorming (BM), Ishikawa diagram, validation technique with Likert scale, arithmetic average, GRA method (Grey Relational Analysis)
[13,14,15,16]	identifying causes of product incompatibility considering the number of their occurrence and proposing the improvement actions	Ishikawa diagram, Pareto–Lorenz analysis
[17]	analysis of the large number of incompatibility causes of product and determining the improvement actions in more precision way, and reducing subjective of experts’ opinion	Ishikawa diagram, 5Why method, FAHP method (Fuzzy Analytic Hierarchy Process)
[18]	method to visualize a gas turbine and to improve the Bowtie risk assessment	Ishikawa diagram, Bowtie analysis
[19]	sequential analysis of errors in assessment in a variety of machine building	three combined Ishikawa diagrams
[20,21]	identification of factors that impact production line downtime in the automotive industry	Ishikawa diagram, 5Why method

**Table 2:** Assessment of the direct influence of second-order causes on the formation of a mechanical seal porosity cluster.

Causes	A1	A2	A3	A4	A5	A6	A7	A8	A9	A10
A1	0	4	1	1	4	1	2	3	1	4
A2	2	0	4	2	3	1	1	2	1	4
A3	4	3	0	3	4	1	1	2	1	3
A4	4	4	4	0	4	1	1	3	1	2
A5	3	3	3	2	0	1	3	2	1	2
A6	4	1	4	1	1	0	4	2	4	3
A7	4	3	3	2	2	1	0	2	1	2
A8	2	2	3	3	4	1	2	0	2	2
A9	1	2	3	2	1	2	1	3	0	1
A10	4	4	3	2	4	1	2	3	1	0

where: A1—wrong gas concentration in the water bath; A2—strong interaction of carbon monoxide with carbon dioxide; A3—not controlling the release of these compounds; A4—low metal temperature; A5—dissolving gas during the casting process; A6—employee mistakes; A7—bad preparation for the product casting process; A8—lack adequate supply of heat distribution centers; A9—incorrectly selected welding parameters; A10—material processing time too long.

**Table 3:** Normalized matrix of direct impact for porosity cluster on the mechanical seal.

Causes	A1	A2	A3	A4	A5	A6	A7	A8	A9	A10
A1	0.00	0.17	0.04	0.04	0.17	0.04	0.08	0.13	0.04	0.17
A2	0.08	0.00	0.17	0.08	0.13	0.04	0.04	0.08	0.04	0.17
A3	0.17	0.13	0.00	0.13	0.17	0.04	0.04	0.08	0.04	0.13
A4	0.17	0.17	0.17	0.00	0.17	0.04	0.04	0.13	0.04	0.08
A5	0.13	0.13	0.13	0.08	0.00	0.04	0.13	0.08	0.04	0.08
A6	0.17	0.04	0.17	0.04	0.04	0.00	0.17	0.08	0.17	0.13
A7	0.17	0.13	0.13	0.08	0.08	0.04	0.00	0.08	0.04	0.08
A8	0.08	0.08	0.13	0.13	0.17	0.04	0.08	0.00	0.08	0.08
A9	0.04	0.08	0.13	0.08	0.04	0.08	0.04	0.13	0.00	0.04
A10	0.17	0.17	0.13	0.08	0.17	0.04	0.08	0.13	0.04	0.00

where: A1—wrong gas concentration in the water bath; A2—strong interaction of carbon monoxide with carbon dioxide; A3—not controlling the release of these compounds; A4—low metal temperature; A5—dissolving gas during the casting process; A6—employee mistakes; A7—bad preparation for the product casting process; A8—lack adequate supply of heat distribution centers; A9—incorrectly selected welding parameters; A10—material processing time too long.

**Table 4:** Structure of total impact for porosity cluster on the mechanical seal.

Causes	A1	A2	A3	A4	A5	A6	A7	A8	A9	A10
A1	0.87	1.01	0.89	0.64	1.07	0.36	0.62	0.81	0.42	0.92
A2	0.94	0.85	0.97	0.66	1.01	0.35	0.56	0.76	0.41	0.90
A3	1.07	1.03	0.89	0.74	1.12	0.38	0.61	0.82	0.44	0.93
A4	1.14	1.13	1.11	0.68	1.20	0.40	0.65	0.90	0.47	0.96
A5	0.95	0.94	0.92	0.65	0.88	0.35	0.63	0.75	0.40	0.82
A6	1.12	1.01	1.09	0.71	1.06	0.36	0.75	0.86	0.58	0.97
A7	0.99	0.95	0.92	0.65	0.97	0.35	0.51	0.75	0.40	0.82
A8	0.95	0.94	0.96	0.71	1.06	0.36	0.61	0.70	0.46	0.84
A9	0.73	0.75	0.78	0.55	0.76	0.33	0.46	0.66	0.30	0.64
A10	1.13	1.13	1.07	0.76	1.19	0.40	0.68	0.90	0.47	0.87

where: A1—wrong gas concentration in the water bath; A2—strong interaction of carbon monoxide with carbon dioxide; A3—not controlling the release of these compounds; A4—low metal temperature; A5—dissolving gas during the casting process; A6—employee mistakes; A7—bad preparation for the product casting process; A8—lack adequate supply of heat distribution centers; A9—incorrectly selected welding parameters; A10—material processing time too long.

**Table 5:** Dependence of causes–effects in the problem of porosity cluster on mechanical seal.

Causes	R	C	R + C	R − C	Identify
A1	7.62	9.9	17.52	−2.28	effect
A2	7.41	9.73	17.14	−2.32	effect
A3	8.03	9.6	17.63	−1.57	effect
A4	8.64	6.77	15.41	1.87	cause
A5	7.30	10.32	17.62	−3.02	effect
A6	8.50	3.65	12.15	4.85	cause
A7	7.31	6.08	13.39	1.23	cause
A8	7.60	7.91	15.51	−0.31	effect
A9	5.95	4.35	10.30	1.60	cause
A10	8.61	8.66	17.27	−0.05	effect

where: A1—wrong gas concentration in the water bath; A2—strong interaction of carbon monoxide with carbon dioxide; A3—not controlling the release of these compounds; A4—low metal temperature; A5—dissolving gas during the casting process; A6—employee mistakes; A7—bad preparation for the product casting process; A8—lack adequate supply of heat distribution centers; A9—incorrectly selected welding parameters; A10—material processing time too long.

**Table 6:** The structure of the total impact with the causes that have a significant mutual influence on the problem of the formation of the mechanical seal porosity cluster.

Causes	A1	A2	A3	A4	A5	A6	A7	A8	A9	A10
A1	0.87	1.01	0.89	0.64	1.07	0.36	0.62	0.81	0.42	0.92
A2	0.94	0.85	0.97	0.66	1.01	0.35	0.56	0.76	0.41	0.90
A3	1.07	1.03	0.89	0.74	1.12	0.38	0.61	0.82	0.44	0.93
A4	1.14	1.13	1.11	0.68	1.20	0.40	0.65	0.90	0.47	0.96
A5	0.95	0.94	0.92	0.65	0.88	0.35	0.63	0.75	0.40	0.82
A6	1.12	1.01	1.09	0.71	1.06	0.36	0.75	0.86	0.58	0.97
A7	0.99	0.95	0.92	0.65	0.97	0.35	0.51	0.75	0.40	0.82
A8	0.95	0.94	0.96	0.71	1.06	0.36	0.61	0.70	0.46	0.84
A9	0.73	0.75	0.78	0.55	0.76	0.33	0.46	0.66	0.30	0.64
A10	1.13	1.13	1.07	0.76	1.19	0.40	0.68	0.90	0.47	0.87

where: A1—wrong gas concentration in the water bath; A2—strong interaction of carbon monoxide with carbon dioxide; A3—not controlling the release of these compounds; A4—low metal temperature; A5—dissolving gas during the casting process; A6—employee mistakes; A7—bad preparation for the product casting process; A8—lack adequate supply of heat distribution centers; A9—incorrectly selected welding parameters; A10—material processing time too long.

**Table 7:** Combinations of integrated significant causes of porosity cluster on the mechanical seal.

Combination Number	Cause and Its Weight	
1	A1 (0.87)	A2 (0.85)
2	A1 (0.87)	A3 (0.89)
3	A1 (0.87)	A5 (0.88)
4	A1 (0.87)	A5 (0.88)
5	A1 (0.87)	A10 (0.87)
6	A2 (0.85)	A3 (0.89)
7	A2 (0.85)	A5 (0.88)
8	A2 (0.85)	A8 (0.70)
9	A2 (0.85)	A10 (0.87)
10	A3 (0.89)	A5 (0.88)
11	A3 (0.89)	A8 (0.70)
12	A3 (0.89)	A10 (0.87)
13	A5 (0.88)	A8 (0.70)
14	A5 (0.88)	A10 (0.87)
15	A8 (0.70)	A10 (0.87)

where: A1—wrong gas concentration in the water bath; A2—strong interaction of carbon monoxide with carbon dioxide; A3—not controlling the release of these compounds; A5—dissolving gas during the casting process; A8—lack adequate supply of heat distribution centers; A10—material processing time too long.

**Table 8:** Ranking supporting dynamic decision-making about actions to improve a mechanical seal with a cluster of porosity.

Combination Number	Cause and Its Weight		Combination Weight	Ranking
10	A3 (0.89)	A5 (0.88)	0.39	1
2	A1 (0.87)	A3 (0.89)	0.39	
12	A3 (0.89)	A10 (0.87)	0.39	
3	A1 (0.87)	A5 (0.88)	0.38	2
4	A1 (0.87)	A5 (0.88)	0.38	
14	A5 (0.88)	A10 (0.87)	0.38	
5	A1 (0.87)	A10 (0.87)	0.38	
6	A2 (0.85)	A3 (0.89)	0.38	
7	A2 (0.85)	A5 (0.88)	0.37	3
1	A1 (0.87)	A2 (0.85)	0.37	
9	A2 (0.85)	A10 (0.87)	0.37	
11	A3 (0.89)	A8 (0.70)	0.31	4
13	A5 (0.88)	A8 (0.70)	0.31	
15	A8 (0.70)	A10 (0.87)	0.30	5
8	A2 (0.85)	A8 (0.70)	0.30	

where: A1—wrong gas concentration in the water bath; A2—strong interaction of carbon monoxide with carbon dioxide; A3—not controlling the release of these compounds; A5—dissolving gas during the casting process; A8—lack adequate supply of heat distribution centers; A10—material processing time too long.

## Data Availability

Not applicable.
